# Proteomic analysis reveals that auxin homeostasis influences the eighth internode length heterosis in maize (*Zea mays*)

**DOI:** 10.1038/s41598-018-23874-6

**Published:** 2018-05-08

**Authors:** Yongqiang Chen, Qingqian Zhou, Runmiao Tian, Zhihui Ma, Xiaofeng Zhao, Jihua Tang, Zhiyuan Fu

**Affiliations:** National Key Laboratory of Wheat and Maize Crop Science/Collaborative Innovation Center of Henan Grain Crops/College of Agronomy, Henan Agricultural University, Zhengzhou, 450002 China

## Abstract

Ear height is an important maize morphological trait that influences plant lodging resistance in the field, and is based on the number and length of internodes under the ear. To explore the effect of internodes on ear height, the internodes under the ear were analysed in four commercial hybrids (Jinsai6850, Zhengdan958, Xundan20, and Yuyu22) from different heterotic groups in China. The eighth internode, which is the third aboveground extended internode, exhibited high-parent or over high-parent heterosis and contributed considerably to ear height. Thus, the proteome of the eighth internode was examined. Sixty-six protein spots with >1.5-fold differences in accumulation (*P* < 0.05) among the four hybrids were identified by mass spectrometry and data analyses. Most of the differentially accumulated proteins exhibited additive accumulation patterns, but with epistatic effects on heterosis performance. Proteins involved in phenylpropanoid and benzoxazinoid metabolic pathways were observed to influence indole-3-acetic acid biosynthesis and polar auxin transport during internode development. Moreover, indole-3-acetic acid content was positively correlated with the eighth internode length, but negatively correlated with the extent of the heterosis of the eighth internode length.

## Introduction

Heterosis refers to a phenomenon in which the survival and performance of a hybrid are superior to those of the genetically distinct parents. As a multigenic complex trait, heterosis has been attributed to dominance, over-dominance, or epistasis based on quantitative genetic theory. Lippman and Zamir^1^ reported that the magnitude and rate of vegetative growth, flowering time, yield, and resistance to biotic and abiotic environmental stresses, which are regulated by many biochemical pathways, influence heterosis to some extent. Additionally, heterosis is trait-dependent, implying that the underlying mechanisms are active within the biological context of specific traits^2,3^. Genome structural variations^4,5^, epigenetic modifications^6^, diverse global expression trends^7,8^, specific protein functions, differential accumulations, and post-transcriptional modifications^7,9–13^ have been associated with the heterosis of specific organs and developmental stages at the molecular level.

Plant lodging in the field not only decreases the final yield, it also hinders the mechanical harvesting of maize (*Zea mays*)^14^. The ratio of ear height to plant height is an important indicator influencing plant stalk lodging resistance, especially during the grain-filling stage^7^. Thus, ear height has been widely studied as an important morphological trait, not only because it influences plant lodging resistance in the field, but also because of its association with high heterosis at the quantitative trait locus (QTL) mapping level. Several stable QTLs for plant height and ear height were detected on chromosomes 1 and 10 in two F_2:3_ populations^9^. Moreover, five QTL clusters for plant height and ear height were identified in 11 recombinant inbred line populations based on 787 single nucleotide polymorphisms (SNPs)^15^. Seven SNPs out of 14,401 were significantly related to ear height according to association mapping results with 159 maize inbred lines, and were co-localised to a QTL region for ear height^16^. Different studies revealed diverse mechanisms related to plant height and ear height heterosis. Some studies confirmed that additive and partial dominance were the major QTL effects on plant height and ear height^17,18^. Meanwhile, Song *et al*.^19^ concluded that dominance, over-dominance, and epistasis contributed to the heterosis of plant height and ear height.

Ear height is based on the basal five or six internodes (depending on the genetic background), which do not elongate and remain underground^4^ as well as the elongating internodes under the ears. The first to sixth internodes normally do not elongate. The internodes begin to elongate from the seventh internode, with the extent of the elongation influenced by various hormones and other factors. Therefore, the number and length of the extended aboveground internodes under the ear determine the final ear height. For any hybrid, superior ear height is reflected by longer internodes rather than a greater number of internodes in comparisons with the inbred lines^5^. The molecular mechanism regulating ear height heterosis should be characterised in detail for a specific internode rather than for the entire plant. Therefore, as an initial study, the sixth to eleventh extended internodes under the ear were phenotyped, and the eighth internode was used for a proteomic analysis. The objectives of this study were to (1) elucidate the contribution of each extended internode to ear height and the associated heterosis and (2) explore the molecular regulatory mechanism related to internode heterosis.

## Methods

### Internode sampling

Four elite Chinese commercial maize hybrids and their parental lines [Jinsai6850 (P2 × A50), Zhengdan958 (Zheng58 × Chang7-2), Xundan20 (Xun9058 × Xun928), and Yuyu22 (Zong3 × Yu87-1)] were used in this study. These hybrids have been classified into the following three main heterotic groups in China: Reid × TangSPT (Zhengdan958 and Xundan20), Lancaster × Lvdahonggu (Jinsai6850), and Zi330 × Tem-tropic I (Yuyu22)^6^. Seeds of the four hybrids and their corresponding parental lines were sowed on the farm of Henan Agricultural University (Zhengzhou, China; E113°42′, N34°48′) in the summer of 2015. The plots consisted of eight rows, each 4 m long, with in-row and inter-row spacings of 25 and 75 cm, respectively. Only the middle rows were sampled to avoid edge effects. The sixth internode is the first aboveground internode because the first five internodes are compressed in the crown under the soil line for these hybrids and inbred lines. To ensure accurate sampling times, the fifth and tenth leaves were marked in the field. Plant height (from soil level to the top of the main tassel branch), ear height (from soil level to the ear), and the lengths of the sixth to eleventh internodes (first to sixth extended aboveground internodes under the ear) were measured for each genotype at the adult plant stage. Measurements were completed with five replicates, with average values calculated for each genotype. Data were analysed with the PROC MIXED model of the SAS 8.0 statistical software package. The eighth internode of four hybrids and their parental lines were sampled when the internodes elongated by about 1 cm (i.e., rapidly growing internodes). Three replicates of internodes were collected (10 plants per biological replicate) and immediately frozen in liquid nitrogen. Samples were stored at −80 °C for a subsequent protein extraction and indole-3-acetic acid (IAA) quantification. At least three replicates of each material were prepared to eliminate probable variations due to biological differences among individual plants. The length of each leaf-inserted internode was measured after flowering.

### Total protein extraction and two-dimensional gel electrophoresis analysis

The collected eighth internodes (1 cm; approximately 1 g) were ground to a powder in liquid nitrogen. Debris were removed, after which an equal amount of ground material for each of the 10 plants per biological replicate was mixed for each genotype. Samples were resuspended with 10 mL pre-cooled trichloroacetic acid (TCA) buffer (10% w/v TCA in acetone with 0.07% β-mercaptoethanol) and vortexed for 2 h at 20 °C. After centrifuging samples at 15,000 × g for 30 min, the supernatant was discarded and the precipitate was rinsed four times with 10 mL chilled buffer (80% acetone with 0.07% β-mercaptoethanol) and a centrifugation at 15,000 × g for 10 min. The final precipitate was freeze-dried under a vacuum, after which the dried protein pellet was resuspended in a buffer [8 M urea, 2 M thiourea, 4% (w/v) CHAPS, and 40 mM dithiothreitol], with 20 µL buffer added for every 1 mg precipitate. Protein concentrations were determined based on a standard curve prepared using bovine serum albumin as a standard. For each biological replicate, 800 μg total protein was analysed by two-dimensional electrophoresis, with three technical replicates. Immobilised pH gradient strips (24 cm, Immobiline DryStrips; Bio Rad, Hercules, CA, USA) with a linear gradient of pH 4–7 were rehydrated with the protein solution for 16 h at 50 V. The isoelectric focusing was completed at 250 V for 30 min (slow), 250 V for 2 h (rapid), 500 V for 2 h (rapid), 1,000 V for 2 h (rapid), 9,000 V for 5 h (linear), 10,000 V for 10 h (rapid), and a constant 500 V for the final 12 h at 20 °C. The strips were then immediately equilibrated with 10 mL Buffer 1 (0.375 M Tris-HCl, pH 8.8, 6 M urea, 20% glycerol, 4% SDS, and 2% DTT) for 10 min followed by 10 mL Buffer 2 (0.375 M Tris-HCl, pH 8.8, 6 M urea, 20% glycerol, 4% SDS, and 2.5% iodoacetamide) for 10 min. The DryStrips were embedded at the top of 12% polyacrylamide gels, and the proteins were separated in the second dimension at a constant voltage of 50 V for 30 min. A constant 200 V was maintained until the electrophoresis was completed.

### Protein analysis by mass spectrometry

After the electrophoretic separation of proteins, the gels were stained with Coomassie brilliant blue G250 and photographed with the PowerLook 2100 XL scanner (UMAX). Protein spots were detected and matched with the default parameters of the “spot detection wizard” function in the PDQuest 8.0 program. The “find spot centers” function was used with default auto-noise smoothing and background subtraction. A Gaussian model was applied to generate a master gel for each image file. All gels were matched to selected reference master gels and normalised in the automated mode followed by manual group correction. The following normalisation parameters were used: “total quantity in valid spots”, “total density in gel image”, “mean of log ratios”, and “local regression model”. Only protein spots that were consistent between replicates were retained. The average intensity of each protein spot, with a fixed effect for genotype, was determined based on an analysis of variance (ANOVA) model. The differences between the hybrid and parental values as well as between the hybrid and mid-parent values were determined. Protein spots with significant differences in the maximum intensities (*P* < 0.05) between the hybrids and their parental lines (i.e., at least 1.5-fold difference) were further analysed by mass spectrometry (MS).

The lists of theoretical peptides for each peptide mass fingerprint combined with tandem MS data were used to search the Viridiplantae database (1,093,002 sequences) maintained by the National Center for Biotechnology Information (downloaded 12 December 2012). The database was searched for homologous sequences using the MASCOT 2.2 program (www.matrixscience.com; Matrix Science). The search criteria were as follows: peptide mass tolerance of 100 ppm; maximum of a single missed tryptic cleavage; fragment mass tolerance of 0.4 Da; carbamidomethylation by cysteine residues as the fixed modification; and oxidation by methionine residues as the dynamic modification. Only proteins with a MASCOT score >60 with 95% confidence and at least two matched peptides were accepted. Unique and top-ranked peptides were selected for filtering homologous proteins. Gene Ontology (GO) functional annotations and the theoretical Mr/pI for the identified proteins were retrieved from http://www.geneontology.org/ and http://www.expasy.ch/tools/pi_tools.html, respectively. Additionally, a Kyoto Encyclopedia of Genes and Genomes (KEGG) pathway enrichment analysis was conducted using Blast2GO.

### Data analysis

Protein spots with average intensities that did not significantly deviate from the mid-parent values (*P* > 0.05) were considered to correspond to additively accumulated proteins. Meanwhile, protein spots with average intensities that significantly deviated from the mid-parent values were considered to correspond to nonadditively accumulated proteins, which were categorised according to the method described by Hoecker *et al*.^10^. We used “ + ” and “−” to indicate that the protein spot intensity for the F_1_ hybrid was similar to that of the high-parent and low-parent, respectively. Additionally, “ ++ ” and “− −” indicated that the protein spot intensity for the F_1_ hybrid was significantly different from that of the high-parent and low-parent, respectively. Furthermore, “ + −” indicated the protein spot intensity identified for the F_1_ hybrid was between that of the mid-parent and high-parent or between that of the mid-parent and low-parent.

### Transcript profiling

Total RNA was extracted from the eighth internode of each hybrid and corresponding inbred lines using the TRIzol reagent (Invitrogen, USA). Primers specific for several genes related to the phenylpropanoid and benzoxazinoid metabolic pathways were designed using Primer Premier 5.0 for a quantitative real-time polymerase chain reaction analysis (qRT-PCR) (Table S1). Primers were also designed for the actin gene, which was used as an internal standard (5′-CGATTGAGCATGGCATTGTCA-3′ and 5′-CCCACTAGCGTACAACGAA-3′). Briefly, the RNA was reverse transcribed to cDNA using M-MLV reverse transcriptase (Promega, USA). The qRT-PCR (25 µL reaction volume) was completed with the SYBR Premix Ex Taq™ Kit (Takara, Dalian, China) and the iQ5 real-time PCR system (BioRad, USA). Experiments were conducted in triplicate, and the average expression level value for each gene was calculated. Differences in the mean expression levels between hybrids were tested by a one-way ANOVA (significance level of 0.05).

### Quantification of indole-3-acetic acid

Three replicates were analysed for each sample under the same conditions. To quantify IAA, each 500-mg sample was homogenised with 1 mL 80% methanol (in H_2_O) and centrifuged at 15,000 × g for 10 min. The subsequent steps of the IAA quantification procedure were completed as previously described^11^.

## Results

### Internode effects on ear height heterosis

**Figure 1:**
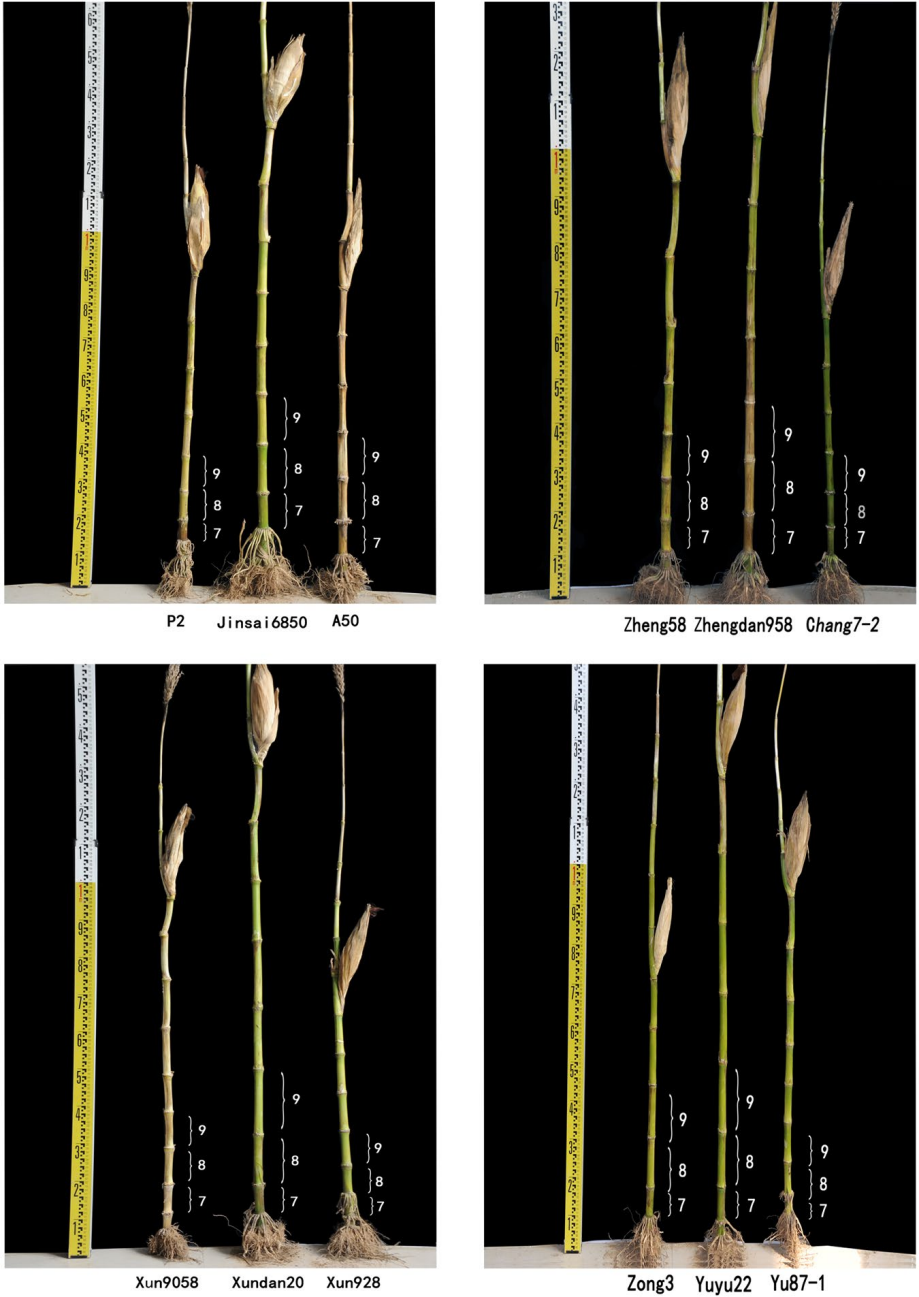
Trait performance and mid-parent heterosis in four hybrids.

**Figure 2:**
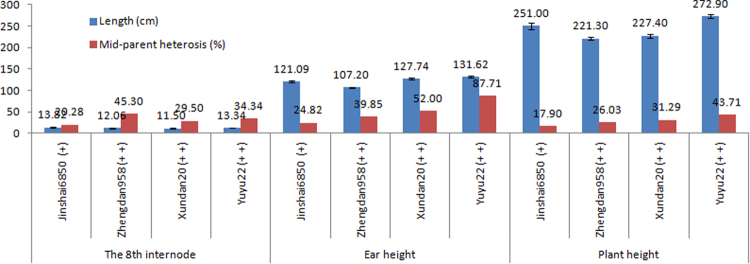
Comparison of internode length and plant height between the hybrids and parental inbred lines.

For the hybrids, ear height, which was determined based on the extended internodes under the ear, was the main factor influencing the heterosis of plant height (from soil level to the top of the main tassel branch), with contributions of 48% for Jinsai6850, Zhengdan958, and Yuyu22, and 56% for Xundan20 (Fig. 1, Table S2). Ear height is also correlated with plant lodging resistance. Thus, characterising the heterosis of extended internodes under the ear may be useful for decreasing the centre of gravity for plants, with potentially important implications for improving plant lodging resistance. The heterosis performance of ear height was mainly influenced by the sixth (2.67–5.68%) to eleventh extended internodes (12.04–14.12%). For the extended internodes, a significant difference (*P* < 0.05) in the contribution to ear height (i.e., proportion of the ear height contributed by the internode length) was detected between the sixth and seventh internodes, and between the seventh and eighth internodes (Table S2). Additionally, there were no significant differences among the eighth to eleventh internodes. Therefore, the eighth internode contributed more to the ear height than the sixth and seventh internodes. During the mature plant stage, the sixth internode was too short (3.52–6.88 cm) to be considered for the final ear height (107.20–131.62 cm) and plant height (221.30–271.90 cm). However, according to the ANOVA results, the eighth internode was the only extended internode that contributed to ear height similarly to the internode above in all hybrids except for Xundan20 (*P* = 0.009), and exhibited high- (Jinsai6850) or over high-parent (Zhengdan958, Xundan20, and Yuyu22) heterosis (Fig. 2; Table S2). An analysis of the hormone contents in neighbouring internodes indicated that the eighth internode underwent less extensive changes than the ninth to eleventh internodes. Moreover, a correlation analysis revealed a relationship between the length of the eighth internode and plant height or ear height for most tested materials, with the extent of the correlation differing among the diverse materials (Table S3). Thus, the proteome of the eighth internode was analysed to clarify the genetic basis of heterosis.

The eighth internode, ear height, and plant height exhibited the following heterosis expression pattern for each hybrid: high-parent expression (+) in Jinsai6850 and over high-parent expression pattern (++) in Zhengdan958, Xundan20, and Yuyu22. The degree of the mid-parent heterosis and high- or over high-parent heterosis decreased from the sixth to eleventh internodes for Zhengdan958 and Jinsai6850. However, the highest mid-parent and high- or over high-parent heterosis for the ninth internode were observed in Xundan20 (57.36 and 52.56%) and Yuyu22 (54.11 and 52.39%) (Fig. 2; Table S2). In other words, the extent of the heterosis was inconsistent with the heterosis performance for the extended internodes, ear height, and plant height.

### Differentially accumulated proteins in the eighth internode

**Figure 3:**
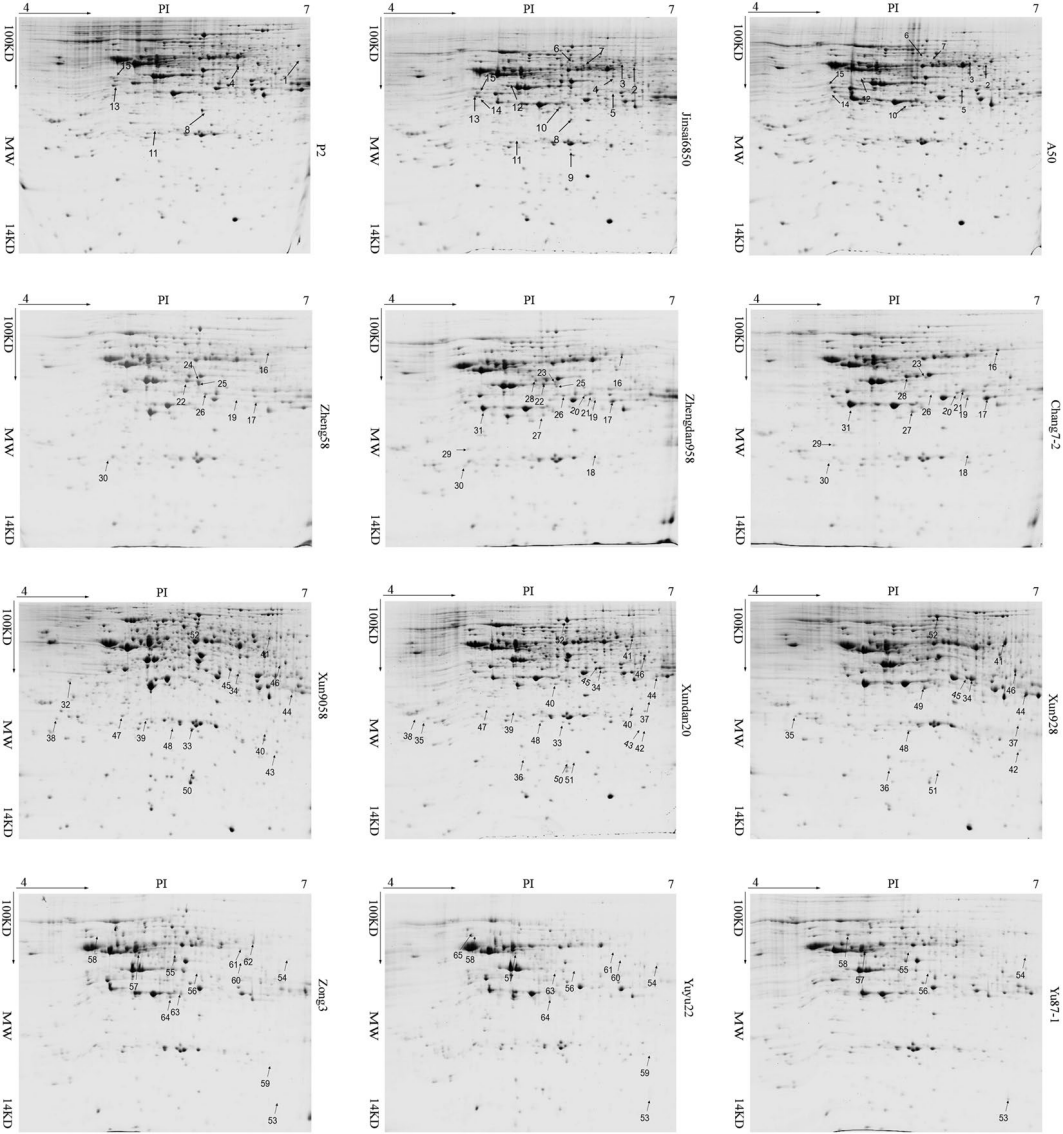
Two-dimensional maps of differentially accumulated proteins in the eighth internode of the hybrids and parental inbred lines. Proteins that accumulated differentially in hybrids and parental inbred lines are numbered.

**Figure 4:**
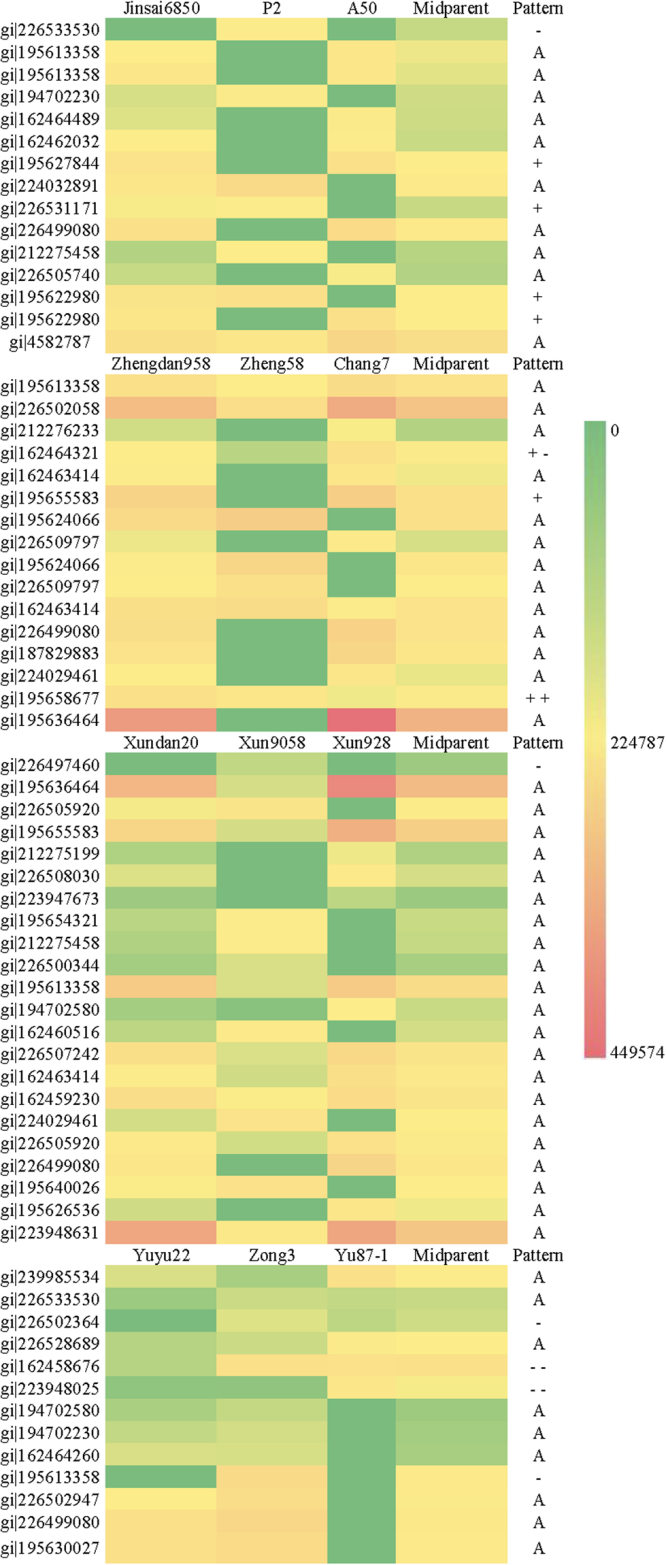
Heat map and abundance patterns of differentially accumulated proteins in the eighth internode of the hybrids and parental lines. Note: Differences in accumulation are presented as a continuous range of colours. Red corresponds to the maximum accumulation (grey value: 449574); green corresponds to the minimum accumulation (grey value: 0); and yellow corresponds to the median accumulation (grey value: 224787).

The proteome of the eighth internode was analysed to identify differentially accumulated proteins among the four hybrids and their parents. A total of 206 (196 and 182), 201 (205 and 198), 180 (229 and 141), and 219 (235 and 232) differentially accumulated protein spots were detected in Jinsai6850 (A50 × P2), Zhengdan958 (Zheng58 × Chang7–2), Xundan20 (Xun9058 × Xun928), and Yuyu22 (Zong3 × Yu87–1), respectively (Fig. 3). Moreover, 15, 16, 22, and 13 protein spots with >1.5-fold difference (*P* < 0.05) were excised from gels and identified by MS, respectively (Fig. 4, Table S4). The majority (54/66) of these protein spots were additively accumulated in the four hybrids, especially in Xundan20 (21/22). The non-additively accumulated protein spots exhibited different patterns in the four hybrids, even for the hybrids in the same heterotic group [i.e., Zhengdan958 (+, ++, and +−) and Xundan20 (−)] (Fig. 4, Table S4). The 66 protein spots (peptides are listed in Table S5) were represented by 46 GenBank accessions. Additionally, some GenBank accessions were identified in the same hybrid twice with different accumulation patterns, likely because of distinct post-translational modifications of the corresponding proteins. GenBank accessions gi|195613358 (GRMZM2G014844_T01; EC 3.2.1.21) and gi|226499080 (GRMZM2G033555_T01; EC1.1.1.219) were identified in all hybrids. However, gi|195613358 accumulated differently in the four hybrids (protein spots 2 and 3 in Jinsai6850, 16 in Zhengdan958, 42 in Xundan20, and 63 in Yuyu22), while the accumulation patterns for gi|226499080 were similar in the four hybrids (protein spot 10 in Jinsai6850, 27 in Zhengdan958, 50 in Xundan20, and 65 in Yuyu22) (Fig. 4, Table S4).

### Functional analysis of the identified proteins

**Figure 5:**
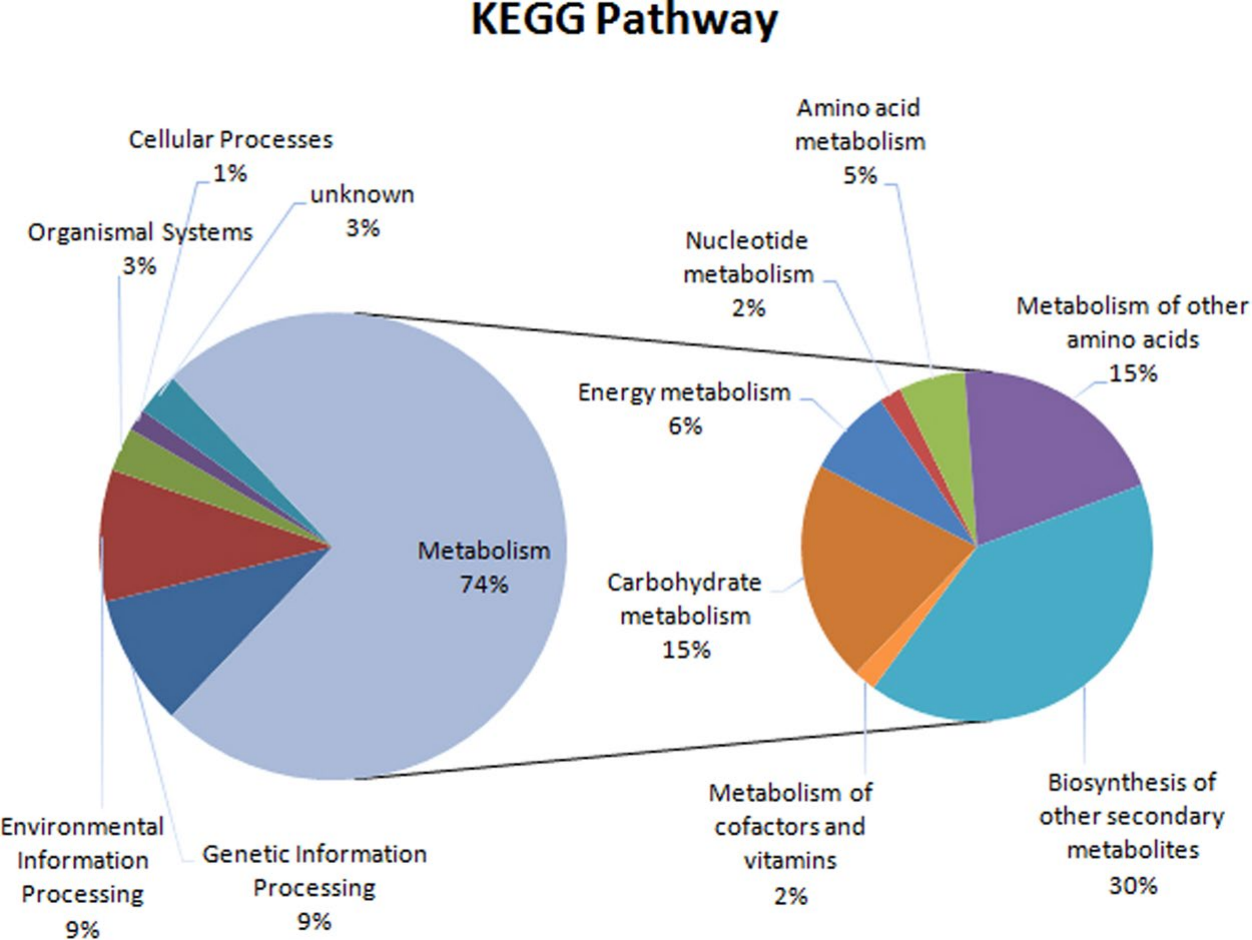
Functional classification of all differentially accumulated proteins in the eighth internode of the hybrids and parental lines. Proteins were classified based on a maize protein database. Note: Proportions of different metabolic activities were calculated based on the number of identified proteins.

The 66 protein spots were classified into the following six GO categories: metabolism, genetic information processing, environmental information processing, organismal systems, cellular processes, and unknown (Fig. 5, Table S6). Metabolism was the largest category, with 74% (49/66) of the differentially accumulated protein spots, implying metabolic activities are critical for internode development and heterosis. The metabolism category was further divided into seven subcategories, including glutathione metabolism, phenylpropanoid metabolism, and benzoxazinoid metabolism, each comprising 10 protein spots. Additionally, two protein spots (gi|195613358 and gi|226499080) related to phenylpropanoid metabolism were differentially accumulated in all hybrids, while four protein spots (gi|162463414, gi|195655583, gi|195624066, and gi|162459230) involved in benzoxazinoid metabolism were differentially accumulated in certain hybrids. Carbohydrate metabolism (10 protein spots), energy metabolism, nucleotide metabolism, amino acid metabolism, and metabolism of cofactors and vitamins were the other metabolism subcategories.

### Relationships among indole-3-acetic acid content, gene expression level, and internode length

The IAA concentrations of the eighth internode were 147.87, 169.24, 253.72, and 328.27 ng/g in Xundan20, Zhengdan958, Yuyu22, and Jinsai6850, respectively (Table 1). The Pearson correlation coefficients indicated a positive correlation between IAA concentration and the eighth internode length (0.976^**^) and ear height (0.212^*^). In contrast, a negative correlation was observed between IAA concentration and the extent of the heterosis of the eighth internode length (−0.654^*^), ear height (−0.166^*^), and plant height (−0.273^**^) (Table 2). These results implied that high IAA levels may induce the elongation of internodes, while inhibiting the heterosis of internode length.

**Table 1:** Indole-3-acetic acid (IAA) content in hybrids and the relative expression levels of key genes influencing IAA biosynthesis and polar distribution.

Hybrids	The 8^th^ internode length (cm)	Relative expression level				IAA (ng/g)	Ear height (cm)	Plant height (cm)	Heterosis degree (Mid-parent)		
		BX2	BX5	FLS	DFR				Ear height (%)	Plant height (%)	The 8^th^ internode length (%)
Xundan20	11.50	1.21	1.11	0.91	1.73	147.87	127.74	227.40	52.0	31.3	29.5
Zhengdan958	12.06	1.00	1.00	1.00	1.00	169.24	107.20	221.30	39.9	26.0	45.3
Yuyu22	13.34	0.95	1.07	0.67	1.65	253.72	131.62	272.90	87.7	43.7	34.3
Jinsai6850	13.82	0.52	0.81	2.62	0.97	328.27	121.09	251.0	24.8	17.9	20.3

**Table 2:** Pearson correlation coefficient analysis of indole-3-acetic acid (IAA) content, gene expression level, eighth internode length, and heterosis extent.

	IAA	Heterosis degree			The 8^th^ internode length	Ear height	Plant height
		The 8^th^ internode	Ear height	Plant height			
IAA		−0.654^*^	−0.166^*^	−0.273^**^	0.976^**^	0.212^*^	0.699
BX2	−0.936^**^						
BX5	−0.790^**^						
FLS	0.746^**^	−0.717^**^	−0.763^*^	−0.827^**^			
DFR	−0.398^**^						

Note: ^*^Significant at the 0.05 level; ^**^Significant at the 0.01 level.

**Figure 6:**
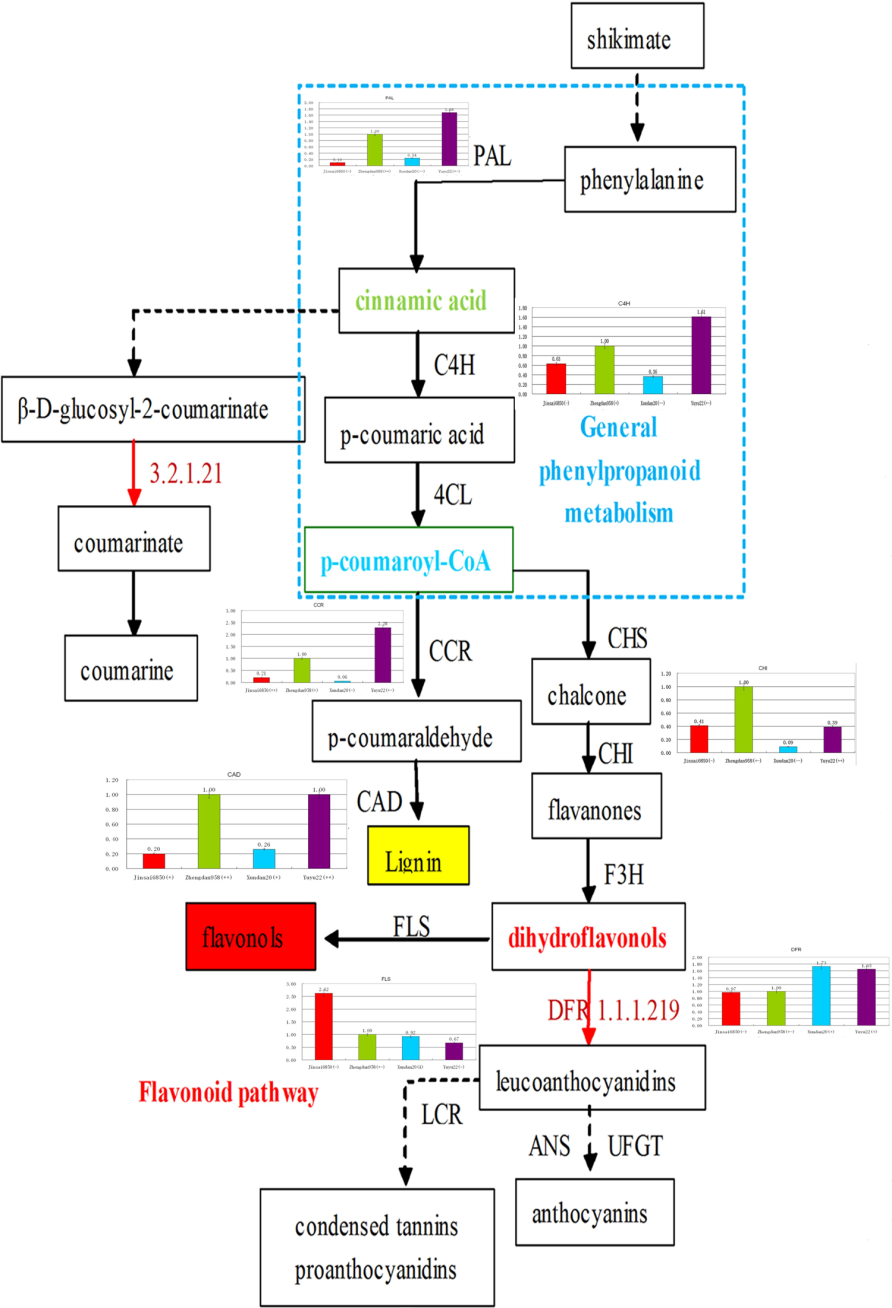
Summary of the phenylpropanoid metabolic pathway as well as the relative expression levels and patterns of key genes in the hybrids. Genes with the associated EC number are provided. Accessions encode the differentially accumulated proteins identified in this study. PAL, phenylalanine ammonia lyase; C4H, cinnamate 4-hydroxylase; CCR, cinnamoyl-CoA reductase; CAD, cinnamyl-alcohol dehydrogenase; FLS, flavonol synthase; DFR, dihydroflavonol 4-reductase; CHS, chalcone synthase; CHI, chalcone isomerase; and F3H, flavanone 3-hydroxylase.

**Figure 7:**
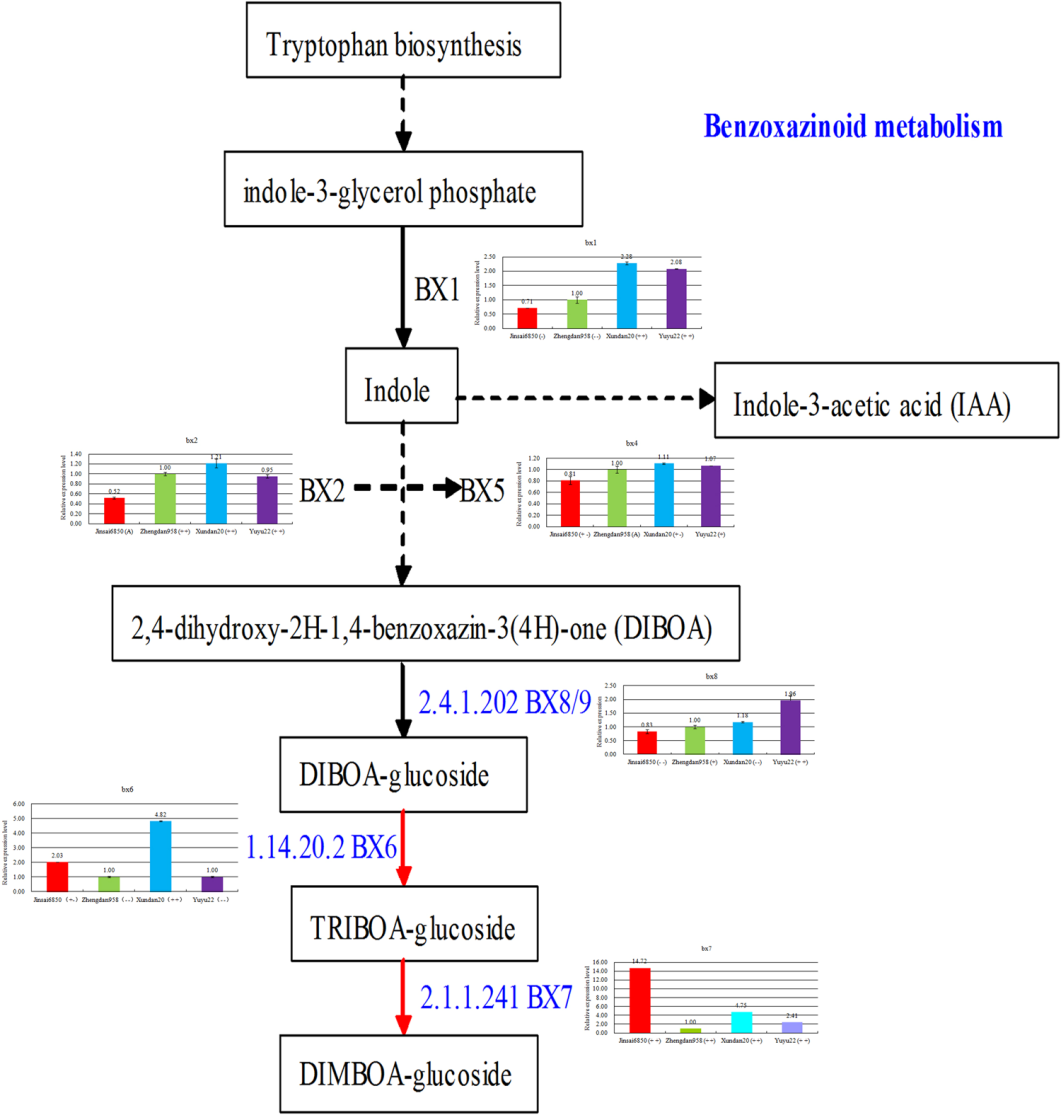
Summary of the benzoxazinoid metabolic pathway as well as the relative expression levels and patterns of key genes in the hybrids. Genes with the associated EC number are provided. Accessions encode the differentially accumulated proteins identified in this study. BX1, benzoxazinless1; BX2 to BX5, cytochrome P450 enzymes; BX8 and BX9, UDP-glucosyltransferases; BX6, dioxygenase; BX7, O-methyltransferase; and DIBOA, 2,4-dihydroxy-2H-1,4-benzoxazin-3(4 H)-one.

The expression profiles of the key genes related to the phenylpropanoid and benzoxazinoid metabolic pathways were examined by qRT-PCR analysis (Figs 6 and 7). In the phenylpropanoid metabolic pathway, phenylalanine ammonia lyase (GRMZM2G170692_T01; EC 4.3.1.24) is the first rate-limiting enzyme. The corresponding gene was highly expressed in Yuyu22 (partial dominance, +−) and Zhengdan958 (above high-parent, ++), and expressed at relatively low levels in Jinsai6850 (low-parent, −) and Xundan20 (below low-parent, − −). The genes encoding cinnamate 4-hydroxylase (GRMZM2G147245_T01; EC 1.14.13.11), cinnamoyl-CoA reductase (GRMZM2G033555_T01; EC 1.2.1.44), and cinnamyl-alcohol dehydrogenase (GRMZM5G844562; EC 1.1.1.195) exhibited similar expression trends in the four hybrids, but were associated with different heterosis patterns (Fig. 6). Dihydroflavonol is the competitive substrate of flavonol synthase (FLS; EC 1.14.11.23) and dihydroflavonol 4-reductase (DFR; EC 1.1.1.219). Our qRT-PCR analysis revealed that the *FLS* and *DFR* expression levels exhibited the opposite trends in the four hybrids, which was consistent with their network functions in the phenylpropanoid metabolic pathway. The IAA content of the eighth internode was positively correlated with the *FLS* expression level (0.746^**^), but negatively correlated with the *DFR* expression level (−0.398^**^) (Table 2). Moreover, the *DFR* expression level and the accumulation of the encoded protein were almost inversely related in the four hybrids (i.e., the highest transcript levels were observed in Xundan20, followed by Yuyu22, Zhengdan958, and Jinsai6850, while the highest protein levels were observed in Zhengdan958, followed by Jinsai6850, Yuyu22, and Xundan20). These results indicated there was a lack of consistency between gene expression and protein accumulation. The IAA biosynthetic pathway and benzoxazinoid metabolic pathway (Fig. 7) compete for free indole. The cytochrome P450 enzyme subfamily (BX2 to BX5) converts indole into benzoxazinoids. The genes encoding these enzymes were highly expressed in Xundan20, and the expression levels were negatively correlated with IAA content. This suggested that IAA biosynthesis was restricted by BX2 and BX5.

## Discussion

### Heterosis performance and differentially accumulated proteins

Dominance, over-dominance, and pseudo over-dominance at a single locus and epistasis at two loci have contributed to the heterosis of different traits according to quantitative genetic analyses^12,13,20,21^. In this study, dominant and over-dominant effects were observed to differentially affect specific internodes in certain hybrids (Table S2). Ear height and plant height were influenced by a dominant effect in Jinsai6850, but an over-dominant effect in Zhengdan958, Xundan20, and Yuyu22. The over-dominant effect on maize plant height was also detected in several other studies^22–24^. The mechanism underlying heterosis has been analysed at transcriptional and translational levels^2^. Additive, dominant, and over-dominant effects on gene expression or protein accumulation differentially contribute to the heterosis of a particular trait at specific developmental stages^2^. The overwhelming additive accumulation of differentially accumulated proteins in the four hybrids was associated with a non-additive effect on the heterosis performance of the eighth internode (Fig. 4, Tables S2 and 4). Similar results were also reported for maize embryos at 6 days after fertilisation^25^, while another study concluded that the moderate transcriptional expression of the *SINGLE FLOWER TRUSS* locus could result in an over-dominant effect on yield^26^. These results proved that an additive interaction at the translational level may lead to a non-additive effect on phenotypic performance due to an epistatic interaction between different proteins.

### Regulation of indole-3-acetic acid affects internode development and heterosis

Certain metabolic and physiological pathways are reportedly relevant for heterosis. Goff^27^ and Ni *et al*.^28^ emphasised the effects of the overall energy-use efficiency and increased energy input for the manifestation of multigenic heterosis. Gibberellins, which are important plant hormones, help regulate stem elongation^29–33^. The metabolic and signalling pathways associated with these hormones are believed to influence plant height heterosis in wheat^5^. This has been supported by similar studies on the role of gibberellins in the heterosis of biomass accumulation in maize^34–36^ and rice^37^. Auxin, which is another important plant hormone, regulates cell elongation, division, differentiation, and morphogenesis^38^. Thus, auxin may be involved in internode development and heterosis.

Indole-3-acetic acid, which is the most potent native auxin^39^, induces most of the auxin effects in intact plants. An appropriate balance between IAA biosynthesis and efflux [polar auxin transport (PAT)] must be maintained in living plants because auxin is toxic at excessive concentrations. Additionally, ethylene accumulates in response to high auxin doses, resulting in inhibited elongation, leaf abscission, and even plant death^40,41^. In this study, the benzoxazinoid and phenylpropanoid metabolic pathways were emphasised because of their effects on IAA biosynthesis and PAT.

For most monocotyledonous plant species, IAA biosynthesis is regulated by indole, which is an intermediate product of the benzoxazinoid pathway^42^. We observed that the *BX2* (−0.936^**^) and *BX5* (−0.790^**^) expression levels were negatively correlated with IAA contents in the eighth internode, but were positively correlated with the eighth internode length. These results implied that balanced IAA metabolism influenced by the benzoxazinoid pathway is important for internode length heterosis. Another secondary metabolite, flavonol, which resembles the synthetic auxin transport inhibitor naphthylphthalamic acid, regulates PAT^43^. Flavonol negatively regulates PAT by competing with auxin efflux carriers, such as PIN and ABCB (PGP proteins) *in vivo*^44–47^. Polar auxin transport refers to the complex and coordinated active transport of auxin molecules from cell to cell throughout the plant. Cell growth (division and expansion) and the generation of auxin gradients throughout the plant require PAT^48,49^. Moreover, PAT allows stems to turn toward light sources (phototropism) and roots to grow in response to gravity (gravitropism), while mediating other tropisms associated with differential cell growth on different sides of an organ^49^. In this study, the expression of the gene encoding FLS, which controls the first committed step of the flavonol branch of phenylpropanoid metabolism, was negatively correlated with the extent of the mid-parent heterosis of ear height and plant height in the four hybrids (Fig. 6, Table 2). These results are consistent with those of previous studies involving *Arabidopsis thaliana*, in which a mutant incapable of synthesising flavonoids exhibited enhanced auxin transport and developmental abnormalities, including an increased number of inflorescences, decreased plant height, increased secondary root development^44^, and delayed gravitropism^47^. Both studies suggested that increased basipetal auxin transport interfered with the formation of an auxin gradient. Additionally, the negative regulation by flavonols was required for plant development and heterosis. Conserved regulators, such as MYB, basic helix-loop-helix, and WD40-type transcription factors, are indirectly involved in the final step of flavonoid translocation through the vacuolar membrane^50^. They also regulate the activation of structural genes, such as *BZ2* in maize^51^, *AN9* in petunia^52^, and *TT19* in *A. thaliana*^53^, which encode glutathione S-transferases^54,55^ involved in cell detoxification. Seven of the identified differentially accumulated protein spots (10.6%) were involved in glutathione metabolism. These results imply that cell detoxification during IAA transport and distribution is critical.

Polar auxin transport was inhibited in Jinsai6850, resulting in the over-accumulation of IAA in the eighth internode, a decrease in the extent of heterosis, and a greater abundance of GST proteins for detoxifying cells. In Yuyu22, only a few flavonols were needed to regulate PAT, which revealed the suitable IAA contents and extensive heterosis. In Zhengdan958 and Yuyu22, which belong to the same heterotic group, IAA content and flavonol regulation exhibited similar trends. Therefore, the heterosis extent and performance were consistent in the eighth internode.

## Conclusions

**Figure 8:**
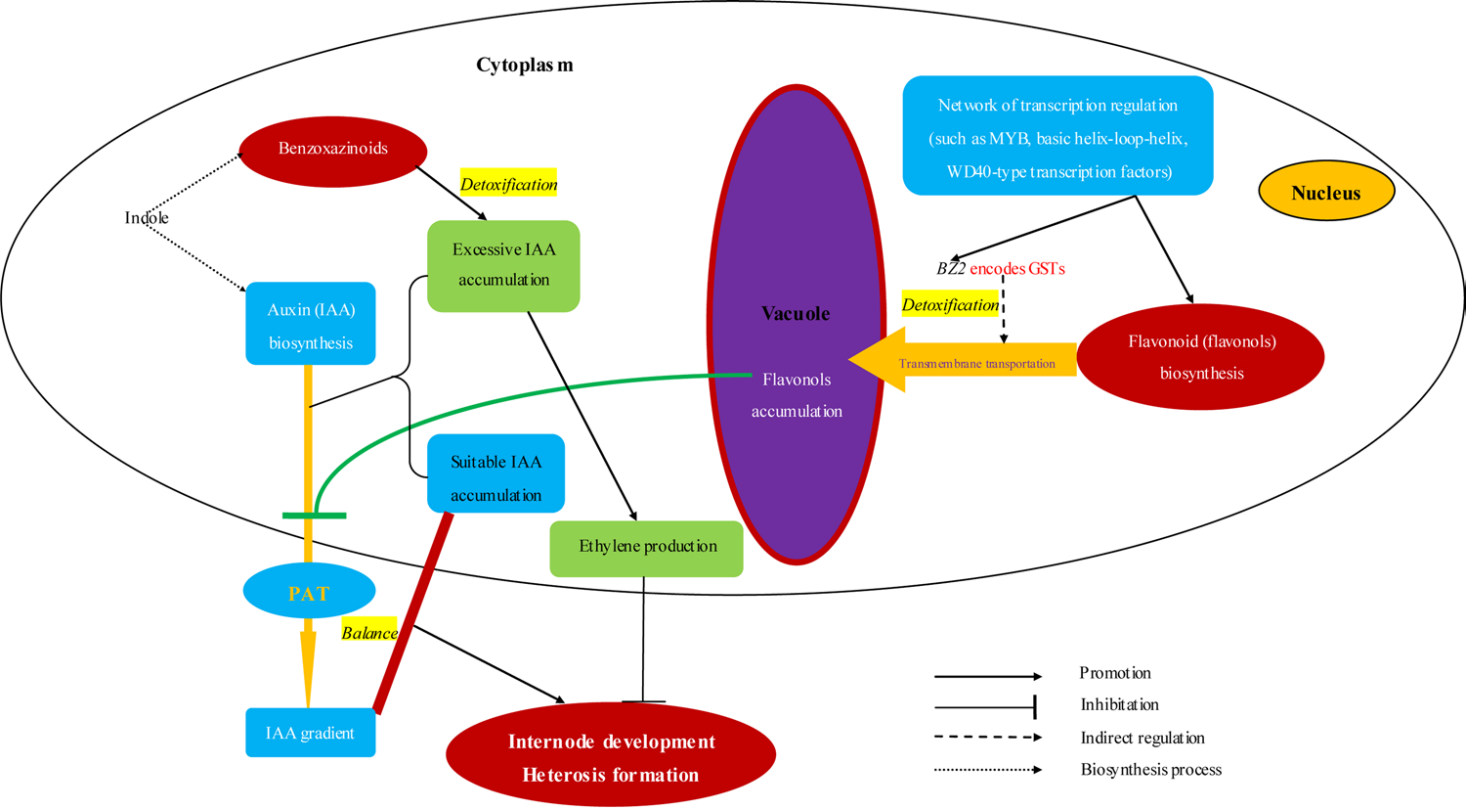
Model of the development and heterosis of internodes. The development and heterosis of internodes are mainly regulated by an auxin gradient, which is the result of a balance between polar auxin transport (PAT) and auxin biosynthesis. In plant cells and organs, PAT is negatively regulated by flavonols, which are biosynthesised in the cytoplasm and stored in the vacuole. Transmembrane transport of flavonols to the vacuole is indirectly regulated by structural genes (e.g., *BZ2*) encoding glutathione S-transferases. Additionally, flavonol biosynthesis and *BZ2* expression are regulated by a network of transcription factors. Once PAT is inhibited, auxin will accumulate in cells and organs to toxic levels. Moreover, excessive auxin concentrations induce ethylene production, which negatively regulates plant development. Thus, detoxification by benzoxazinoid metabolites and the competition for indole between the indole-3-acetic acid biosynthesis pathway and the benzoxazinoid metabolic pathway are required to protect cells and organs. Therefore, flavonols, which are synthesised in the phenylpropanoid metabolic pathway, and benzoxazinoids are important regulators of the development and heterosis of internodes *via* a complex network of key structural genes and transcription factors.

In different heterotic combinations, additive effects at a single locus and epistatic effects at two loci are primarily responsible for the non-additive effects on the heterosis performance of the eighth internode length and ear height. Regarding epistatic interactions, an integrated metabolic pathway helps regulate each intermediate. A regulatory network involving IAA biosynthesis and PAT, which can be affected by the phenylpropanoid and benzoxazinoid metabolic pathways, should be included in the molecular mechanism underlying the development of internode heterosis (Fig. 8).

## Electronic supplementary material

Table S1

Table S2

Table S3

Table S4

Table S5

Table S6
